# Do Intensive Care Data on Respiratory Infections Reflect Influenza Epidemics?

**DOI:** 10.1371/journal.pone.0083854

**Published:** 2013-12-31

**Authors:** Antonie Koetsier, Liselotte van Asten, Frederika Dijkstra, Wim van der Hoek, Bianca E. Snijders, Cees C. van den Wijngaard, Hendriek C. Boshuizen, Gé A. Donker, Dylan W. de Lange, Nicolette F. de Keizer, Niels Peek

**Affiliations:** 1 Department of Medical Informatics, Academic Medical Center, Amsterdam, The Netherlands; 2 Centre for Infectious Disease Control, National Institute for Public Health and the Environment, Bilthoven, The Netherlands; 3 Department of Statistics, Mathematical Modeling and Data Logistics, National Institute for Public Health and the Environment, Bilthoven, The Netherlands; 4 NIVEL, Netherlands Institute for Health Services Research, Dutch Sentinel General Practice Network, Utrecht, The Netherlands; 5 Department of Intensive Care, University Medical Center, Utrecht, The Netherlands; The Ohio State University, United States of America

## Abstract

**Objectives:**

Severe influenza can lead to Intensive Care Unit (ICU) admission. We explored whether ICU data reflect influenza like illness (ILI) activity in the general population, and whether ICU respiratory infections can predict influenza epidemics.

**Methods:**

We calculated the time lag and correlation between ILI incidence (from ILI sentinel surveillance, based on general practitioners (GP) consultations) and percentages of ICU admissions with a respiratory infection (from the Dutch National Intensive Care Registry) over the years 2003–2011. In addition, ICU data of the first three years was used to build three regression models to predict the start and end of influenza epidemics in the years thereafter, one to three weeks ahead. The predicted start and end of influenza epidemics were compared with observed start and end of such epidemics according to the incidence of ILI.

**Results:**

Peaks in respiratory ICU admissions lasted longer than peaks in ILI incidence rates. Increases in ICU admissions occurred on average two days earlier compared to ILI. Predicting influenza epidemics one, two, or three weeks ahead yielded positive predictive values ranging from 0.52 to 0.78, and sensitivities from 0.34 to 0.51.

**Conclusions:**

ICU data was associated with ILI activity, with increases in ICU data often occurring earlier and for a longer time period. However, in the Netherlands, predicting influenza epidemics in the general population using ICU data was imprecise, with low positive predictive values and sensitivities.

## Introduction

A limitation of current influenza surveillance systems is that timely information on severe influenza illness requiring hospital admission is not available. Influenza surveillance in most countries is based upon sentinel general practitioners (GP) networks and the collected information on influenza like illness (ILI) is dependent on the health care seeking behavior of the general population, which can fluctuate with for example media attention. The implementation of a hospital based surveillance system for severe acute respiratory infection (SARI) is now promoted by the World Health Organization (WHO) and European Centre for Disease Prevention and Control (ECDC) as a public health priority worldwide, both for routine surveillance and for preparedness [1], [2], such as in the case of the Middle East respiratory syndrome coronavirus (MERS-CoV). SARI surveillance can focus on admissions for respiratory infections in general hospital wards or in intensive care units (ICU). During the pandemic period, hospitalization for laboratory confirmed Influenza A(H1N1)pdm09 infection was notifiable in the Netherlands. In the season 2009/2010 as well as in the season 2010/2011, ILI incidence as measured by GP sentinel practices, reached the epidemic threshold of 5.1 consultations per 10.000 enlisted patients at a time when already more than 100 patients had been hospitalized, with several ICU admissions and deaths from laboratory confirmed Influenza (National Institute for Public Health and the Environment, unpublished surveillance data).

Hospital admission for influenza is not notifiable anymore and in the Netherlands SARI cases are not routinely collected. An alternative source of information could be the Dutch National Intensive Care Evaluation registry (NICE) [3], wherein diagnostic, and physiologic information from the first 24 hours of adult ICU admissions, as well as length of stay and in-hospital mortality of all ICU patients are registered. Patients are admitted to the ICU if they have a high severity of illness, and require constant monitoring of their vital functions, regardless of their expected outcome. Respiratory infections, such as pneumonia, are among the most common conditions for which patients are admitted to the ICU. In the early onset of an influenza epidemic, patients with multiple comorbidities are more likely to develop more severe influenza related diseases like pneumonia. They possibly get submitted to the ICU before there is an epidemic in the general population [4]. Therefore, increases in the number of admissions at the ICU with a respiratory infection can possibly occur before a detectable increase in ILI incidence in the GP sentinel network.

In this study we explore whether ICU data on respiratory infections reflect ILI activity in the general population, and which relevant time lag exists between both data sources. Additionally we assessed whether ICU data can predict ILI defined influenza epidemics (further referred to as influenza epidemics).

## Methods

In the Netherlands, patients that consult the GPs with symptoms of ILI are reported on a weekly basis to the Continuous Morbidity Registration Sentinel General Practice Network (further referred to as sentinel GP registry), covering 0.8% of the Dutch population being nationally representative by age, gender, regional distribution, and population density [4]. The sentinel GP data consists of the weekly number of patients presenting with ILI at the GP. In 2009, the registry had 42 participating GP practices with 59 GPs covering a population of approximately 130,000 patients [4]. ILI was defined according to the criteria of Pel [5], by (1) acute onset, with prodromal stadium of three to four days, (2) a temperature increase to at least 38 degrees Celsius, and (3) at least one of the following symptoms: cough, nasal congestion, raw throat, frontal headache, retrosternal pain, and body aches. Incidence of ILI is calculated on a weekly basis, using number of patients registered at the reporting GP practices as the denominator. This is acceptable as almost every person in the Netherlands is registered with a GP. In the Netherlands, an influenza epidemic is defined as more than 5.1 patients with ILI per 10,000 inhabitants per week consulting the GP for at least two consecutive weeks [6], combined with influenza A virus isolation from laboratory samples. Data quality is assured by training of GPs for data entry, and a pop-up appearing in the system when an ILI diagnosis is entered reminding the GP to register the ILI case in the sentinel GP registry.

The NICE registry was founded in 1996, with initially six participating ICUs. In 2003 this number had grown to 33 and in 2011 85 ICUs participated covering approximately 90% of the Dutch adult ICUs. For each admission, among other items, the Acute Physiology and Chronic Health Evaluation II (APACHE II) [7] reason for ICU admission diagnosis is registered and sent to the NICE registry on a monthly basis. Upon receipt, the data is usually entered into the NICE registry database and available for participants within one day. There is currently no distinct variable describing whether an ICU patient has influenza like illness or was diagnosed with an influenza virus infection, or receives antiviral drugs. Therefore, we defined an ICU admission with a possible SARI (further referred to as ICU admission with respiratory infection) when the following four criteria were met: (1) the patient was admitted to the hospital less than two days before ICU admission, (2) there was a medical (non-surgical) reason for admittance, (3) the ICU admission was not a readmission to the ICU within the hospitalized period, and (4) the APACHE II reason for admission was ‘Respiratory Infection. We used the percentage of ICU admissions with a respiratory infection (relative to the total number of medical ICU admissions), instead of the absolute number of ICU admissions to adjust for the growing number of NICE registry participants throughout the study period. Thus our study dataset included study year, week number, number of patients with ILI, population size of reporting GPs, number of ICU admissions with respiratory infection, and number of medical ICU admissions. Data quality is assured by regular on site visits of the ICUs [8].

In this study, we used weekly time series of patients presenting with ILI from the sentinel GP registry and ICU admissions of respiratory nature from the NICE registry. In the NICE registry, few ICUs were participating in the years 2000 until 2002, leading to large variations in the percentage of ICU admissions with respiratory infections. Therefore, from both registries, we used data from 2003 through 2011. As influenza generally occurs between week 40 and week 20 of the subsequent year [9], we defined an influenza year (i.e. season) from July 1st until June 30th the next year, thereby having ten influenza years in our dataset.

### Statistical Analysis – Association between ICU and ILI Data

To explore the association between the weekly incidence of ILI patients and the percentage of ICU admissions with a respiratory infection, we plotted per week the percentage of ICU admissions with a respiratory infection and the incidence of ILI over the period January 1, 2003, through December 31, 2011. In addition, we performed a Generalized Estimating Equations (GEE) [10] additive Poisson regression analysis [11] using an autoregressive working correlation matrix over this time period. The dependent variable was weekly incidence of patients with ILI, and the independent variables were chronological week number, percentage of ICU admissions with respiratory infection in the current week, and one to five weeks before the current week and one to five weeks after the current week, and sine and cosine terms to adjust for seasonality [12]. The sine term was sin(k2πt/T) and the cosine was cos(k2πt/T), where k is a constant with values 1 (yearly seasonality) or 2 (half year seasonality), t is current week number, and T is total number of weeks in the specific influenza year, e.g. 52 weeks (years 2004 and 2009 had 53 weeks, and the cases were added to week 52). We adjusted for autocorrelation in the residuals, where the unit of clustering was influenza year. We calculated the average time lag between the sentinel GP and ICU data by computing the weighted average of the time lag in weeks (−5, …, 0, …, +5), using the corresponding regression coefficients as weighting factors. The R-squared (*R^2^*) value based on the deviance residuals [13] was also calculated.

### Statistical Analysis – Predicting Influenza Epidemics

To assess the possibility of using ICU data for predicting influenza epidemics, we used a subset of three years of training data to develop three GEE models with the same characteristics as the aforementioned model, to predict the incidence of ILI patients one to three weeks ahead. In each of these models, independent variables were removed by pseudo stepwise selection [14], using a fixed scheme for removal. We first considered the time trend (chronological week number) for removal, then seasonal terms, and finally time lagged percentage of ICU admissions with respiratory infection. In order for the final models to be useful in surveillance, the following restrictions also applied: (1) the percentages of ICU admissions with respiratory infection one to five weeks after the current week were not included as they are unavailable and useless for prospective surveillance, (2) the percentage of ICU admissions with respiratory infection in the current week cannot be removed from the model, (3) additional variables of lagged ICU admissions should correspond to a range of subsequent weeks (e.g., one and two weeks before the current week, not one and three weeks before the current week), and (4) a seasonal term is always a combination of a sine and cosine function. We used data of the first three influenza years (January 1, 2003, through June 30, 2005) to generate the final model for predicting the incidence of ILI one week ahead. To accomplish this, we varied the decay factor *λ* of the full model, giving weeks further back in time exponentially lower weights, from 0.97 to 1.00 with increments of 0.005, resulting in seven candidate models. Variable selection for these seven models was performed with pseudo stepwise selection, with the Quasi-likelihood Information Criterion (QIC) [15] as performance measure. To determine the optimal value of *λ*, 10-fold cross validation [16] was performed for the seven candidate models using the same three influenza years that they were built with. The model with the best *R^2^* value was selected as final model. The above steps were repeated for variable selection of the models predicting ILI two and three weeks ahead.

Using the final models based on the 3 training years of data we started predicting the incidence of ILI patients week by week starting from the fourth influenza year in our dataset (July 1, 2005) onward. Before predicting each successive week, the model parameters were recalculated with an updated dataset that included the data of the previous week to make the model dynamic. We continued updating the model parameters week by week, until all remaining seven influenza years were predicted. From the fourth influenza year onward, the predicted incidence of ILI patients was plotted together with the observed incidence of ILI patients. We used the same threshold as the ILI sentinel surveillance to define an influenza epidemic in the predicted ILI numbers (incidence > = 5.1 ILI patients per 10,000 inhabitants for at least two consecutive weeks). We compared the predicted epidemic weeks using ICU data with observed epidemic weeks based on ILI data. Accordingly, we calculated the positive predictive value (PPV), and sensitivity of the predicted epidemic weeks on a weekly basis. For comparison, we also predicted the weekly incidence of ILI with models that used only seasonal terms and auto-regressive ILI variables, but excluded ICU data. These models were also created with pseudo stepwise selection. The resulting PPV and sensitivity were also calculated.

The statistical analyses were performed using the statistical package R, version 2.15.1 (http://www.r-project.org/; Vienna, Austria).

## Results

In the period January 1, 2003, through December 31, 2011 there were a total of 477,422 ICU admissions, of which 133,615 (28.0%) had a medical (non-surgical) reason for admittance. Of the medical ICU admissions, 17,786 (13.3%) were for a respiratory infection (Table 1). The incidence of ILI was on average 3.2 per 10,000, with a standard deviation of 2.8, and a minimum of 0.1 per 10,000 and a maximum of 17.5 per 10,000. There were nine epidemics, consisting of 72 weeks in total, and an average length of eight weeks. On average, 43 GPs supplied data on ILI.

**Table 1:** Number of participating Intensive Care Units (ICUs), number and percentage of medical ICU admissions for respiratory infections and gender and age distribution.

Year	Participating ICUs	ICU patients with respiratory infections (%)	Gender, male (%)	Age, mean (SD)
2003	33	750 (12.8)	431 (57.5)	63.82 (15.8)
2004	36	778 (10.8)	452 (58.1)	63.00 (17.00)
2005	45	1140 (12.8)	657 (57.6)	63.47 (15.8)
2006	56	1585 (13.9)	954 (60.2)	63.84 (16.6)
2007	62	1876 (12.9)	1169 (62.3)	64.14 (16.1)
2008	68	2202 (13.4)	1245 (56.5)	64.46 (15.9)
2009	77	2974 (14.1)	1743 (58.6)	64.43 (16.0)
2010	81	3173 (13.4)	1885 (59.4)	64.84 (15.9)
2011	85	3308 (13.6)	1974 (59.7)	64.19 (16.2)

### Association between ICU and ILI Data

**Figure 1:**
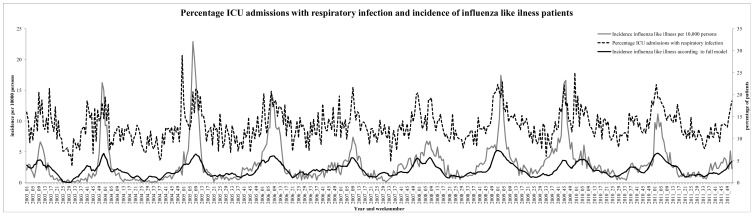
Percentage of medical Intensive Care Unit (ICU) admission with respiratory infection, and incidence of Influenza Like Illness (ILI) cases in the period 2003–2011. Incidence of ILI is plotted per 10,000 population per week. The ILI according to the full model is also plotted.

Both the incidence in ILI and percentage of ICU admissions with a respiratory infection show a similar timing of seasonal peaks (Figure 1). The amplitude of the yearly peaks in the ICU data were relatively lower than ILI peaks, and often lasted for a longer time period. Increases in the incidence of ILI showed a yearly pattern and increased in a smoother pattern compared to ICU data. While trends were roughly comparable, they differed in some instances since peaks in ICU data occasionally occurred when ILI increases were absent in the general population.

The association between the different lags of ICU admissions and ILI incidence is shown in Table 2 (assessed using GEE additive Poisson regression analysis). The *R^2^* value of the full model was 0.58. Of each variable, the contribution to the *R^2^* of the full model is also shown, which is the *R^2^* value of the full model minus the *R^2^* of the model with the corresponding variable omitted. Figure 1 also shows the predicted ILI incidence (black line) according to the full model. Statistically significant time lags were percentage of ICU admissions with respiratory infection were one week before, current week, one week after, two weeks after, and four weeks after current ILI incidence. The time lags mostly associated with increases in ILI incidence one week before and in the current week, with coefficients of 0.12 and 0.11. For example, if the percentage of ICU admissions with a respiratory infection in the current week increased by one percent, then the incidence of ILI in the general population increased by 0.11 per 10,000 population. According to the contribution to *R^2^*, also ICU admissions one week later was strongly associated with current ILI (coefficient of 0.08). There is no linear time trend present, but seasonality exists in the data reflected by a half year sine function (sine term with k = 2) with a p-value of <0.01 and a large contribution to *R^2^*. Looking at Figure 1, a yearly time trend would be expected but is now partly reflected in the different ICU time lagged variables. Using the coefficients of the time lags in Table 2, the average of the weighted relative week numbers was −0.24 weeks implying that the increase in percentage of ICU admissions with a respiratory infection was on average 1.68 days earlier than the increase in ILI incidence.

**Table 2:** GEE additive Poisson regression analysis for assessing the relation between the weekly incidence of Influenza Like Illness and percentage of medical Intensive Care Unit (ICU) admissions with respiratory infection.

Variable	Coefficient	p-value	Contribution to R^2^
Chronological week number	0.00	0.84	0.00
percentage of ICU admissions with respiratory infection five weeks before	0.01	0.62	0.00
percentage of ICU admissions with respiratory infection four weeks before	0.01	0.78	0.00
percentage of ICU admissions with respiratory infection three weeks before	0.03	0.33	0.01
percentage of ICU admissions with respiratory infection two weeks before	0.04	0.21	0.01
percentage of ICU admissions with respiratory infection one week before	0.12	<0.01	0.03
percentage of ICU admissions with respiratory infection in current week	0.11	<0.01	0.03
percentage of ICU admissions with respiratory infection one week after	0.08	<0.01	0.03
percentage of ICU admissions with respiratory infection two weeks after	0.05	0.03	0.00
percentage of ICU admissions with respiratory infection three weeks after	0.01	0.52	–0.01
percentage of ICU admissions with respiratory infection four weeks after	0.02	0.04	0.00
percentage of ICU admissions with respiratory infection five weeks after	–0.01	0.30	0.00
Sine term with k = 1	–0.08	0.87	0.00
Cosine term with k = 1	–0.40	0.22	0.01
Sine term with k = 2	0.52	<0.01	0.08
Cosine term with k = 2	–0.08	0.72	0.00

The contribution to R^2^ of each variable is also shown, this is the R^2^ value of the full model minus the R^2^ of the model with the variable omitted.

### Predicting Influenza Epidemics

**Figure 2:**
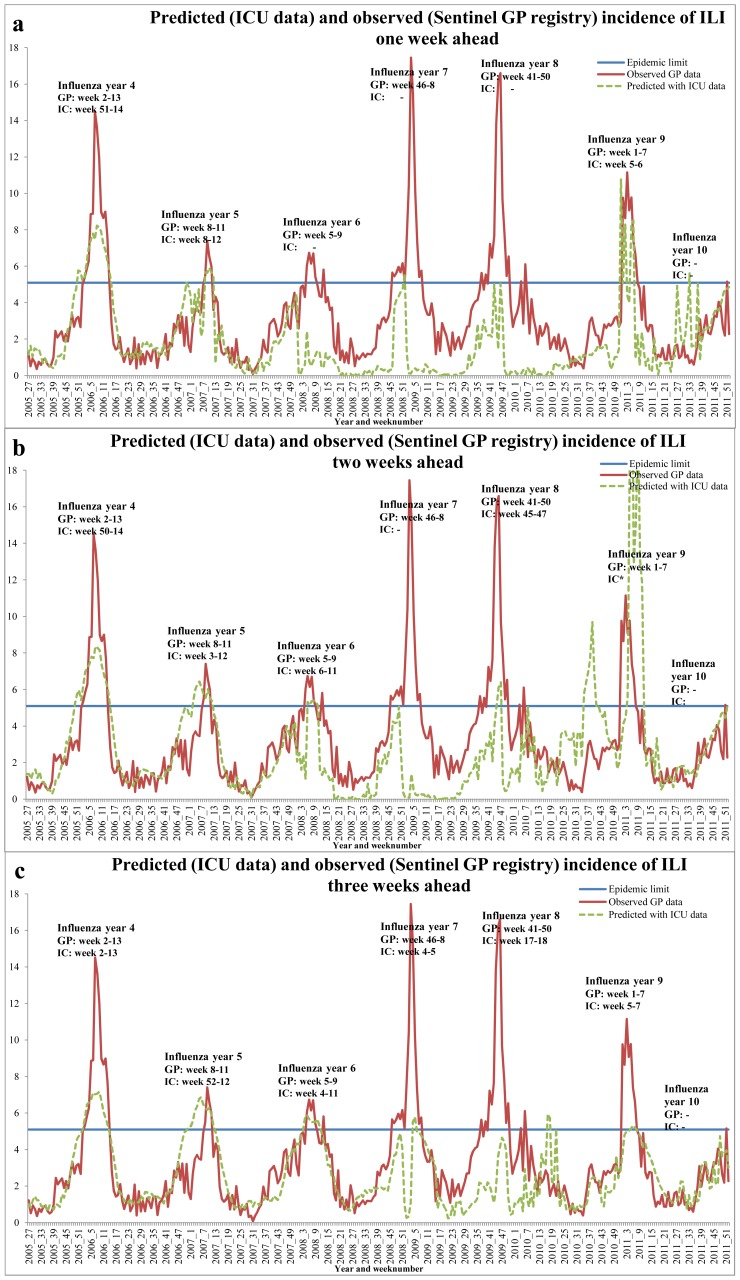
Observed incidence of Influenza Like Illness (ILI) according to the Sentinel General Practitioners registry (sentinel GP registry) and predicted according to Intensive Care Unit (ICU) data. Weeks where an influenza epidemic was detected are also shown. Figure (a), (b), and (c) predict one to three weeks ahead. Weeks marked with an *-sign indicate multiple detections of an influenza epidemic in one influenza year.

For our second research question, whether ICU data can predict ILI incidence ahead in time, we generated three GEE models predicting the incidence of ILI patients one to three weeks ahead using ICU data. Table 3 shows these three different GEE models, and their PPV and sensitivity. Predicting two weeks ahead yields the largest sensitivity of 0.51 and predicting one week ahead has the largest PPV of 0.78. For comparison, models using only auto-regressive ILI variables and seasonal terms, showed the sensitivity to range between 0.21–0.22 and the PPV between 0.31–0.37. Figure 2 shows three figures plotting the predicted incidence of ILI patients one to three weeks ahead versus the actually observed ILI incidence. The epidemic threshold of 5.1 patients (or more) with ILI per 10,000 population is plotted and the weeks in which an influenza epidemic occurred according to the predicted versus the actual data is shown.

**Table 3:** GEE model covariates and decay factor λ, giving historic data less weight, used for predicting one to three weeks ahead.

Prediction	Model covariates	PPV	Sensitivity	*λ*
Oneweekahead	Percentage of ICU admissions with respiratory infectioncurrent week+one week before+two weeks before+threeweeks before+Cosine and Sine term with k = 1+ Cosineand Sine term with k = 2+ Chronological week number	0.78	0.34	0.980
Twoweeksahead	Same as one week ahead	0.52	0.51	0.980
Threeweeksahead	Same as one week ahead, except ‘Percentage of ICUadmissions with respiratory infection four and five weeksbefore’ is also included	0.65	0.49	0.995

The performance of the GEE models in predicting the start, end and length of an influenza epidemic is expressed by the positive predictive value (PPV), and sensitivity (n = 338 weeks) based on comparing the signals for an epidemic predicted with intensive care unit (ICU) data with the reference standard from the observed Influenza Like Illness data.

Predicting one week ahead detected three of the six epidemics, of which two were longer and one shorter according to the ICU-based predictions. One epidemic was predicted earlier, one at the same time and one later. Using ICU data to predict two weeks ahead resulted in five of the six epidemics detected, one is missed, and one false epidemic is predicted in the autumn of 2010 (which was not present in the ILI data). According to the ICU data, the predicted epidemics were longer in time except one. Besides, most predictions were shifted in time compared to the actual occurrence: two epidemics were predicted earlier and three later. When predicting three weeks ahead all six epidemics were detected, however four were shorter in length, one longer, and one had the same length, again shifted in time: two epidemics were predicted earlier, three later, and one at the same time with ICU data.

## Discussion

The study showed that the percentage of medical ICU admissions for respiratory infection was associated with weekly incidence of ILI in the current week, and with one week positive and negative time lag. An increase in the percentage of ICU admissions for respiratory infections on average preceded the increase in the incidence of ILI (GP data) by 1.68 days, implying that before an epidemic the severely ill influenza cases get admitted to the ICU. Despite this precedence, our analyses showed that with the current models ICU data do not accurately predict influenza epidemics in the general population, but including ICU data showed an improvement in sensitivity and PPV compared to only including auto-regressive ILI variables and seasonal terms.

In our study we built three additive Poisson GEE regression models with ICU data to predict the incidence of ILI patients, thereby detecting influenza epidemics and aimed at detecting opportunities for enhancing the current national surveillance method. Previous studies also aimed at enhancing their current surveillance of influenza epidemics, using laboratory or hospital data. Steiner et al. [17] used an exponentially weighted moving average control chart to enhance and automate influenza epidemic detection. Weekly laboratory notifications data of seven years were used instead of the ILI data that we studied. The predicted influenza epidemics were compared to retrospective inspection of the same notification data by epidemiologists. The predictions were, just like our study, not the same as their reference data. However in their study there was a maximum of one week difference only, except for one year where there was a difference of eight weeks. A study by Closas et al. [18] used a Kolmogorov-Smirnov test with virologic laboratory data of five years to detect influenza epidemics. The test provides a binary signal indicating epidemic activity and a quantitative measure of its confidence. They sequentially updated the test as new data became available. The results differed one to nine weeks with the retrospective data of the sentinel network, which is comparable to our results. Google Flu Trends also aimed to detect influenza epidemics, but overestimated peak influenza levels [19] whilst our study underestimates peak influenza levels. These methods complement the current surveillance networks, but cannot replace them.

A study by van den Wijngaard et al. [20] did not aim to predict influenza epidemics, but instead explored whether excesses in influenza severity per season can be detected by combining GP, hospital, laboratory, and mortality data (7 years of data). Their finding was that combining these data sources is of added value, allowing for better understanding of increases in severe morbidity and mortality due to influenza infections. Also from our data we see that trends in ICU related SARI differ from the trends of ILI in the general population and may thus be of value in offering additional information on severity of influenza seasons which need to be explored further. However, both respiratory ICU admissions and ILI in the general population are not necessarily caused by influenza alone. Microbiological laboratory results would provide better insight but to date, these data are not available at the ICU patient level.

The major strength of our study is that we had access to two large historical datasets from the NICE registry and the sentinel GP registry. This allowed us to retrospectively analyze ten influenza years of data, which, to our knowledge, is a longer time period than in comparable studies. A second strength of our study is that we used GEE in our additive Poisson model, thereby correcting for correlations between weeks. The last strength of our method is that for each additional week we sequentially updated the coefficients of the covariates in the model used for prediction of ILI, adding a decay factor giving historic data less weight, and adjusted our models for seasonal changes. With these adjustments, our models always incorporated the most recent information on ICU and ILI trends.

A limitation of the NICE registry data is that there is no distinct variable describing whether a patient has an influenza like illness or whether an patients has been diagnosed with an influenza virus infection. Furthermore it only contains adult patients thus representing an older population compared to the ILI surveillance which also includes children. We extracted admissions with a medical respiratory infection, admitted to the ICU within two days after hospital admission, and excluding readmissions. These admissions represent community-acquired respiratory infections and, therefore, included influenza virus infections. Additionally, the data of the sentinel GP registry is weekly updated, whereas the NICE registry is updated on a monthly basis. This frequency is developed because outcome data, e.g. mortality, is measured at hospital discharge. For sentinel purposes this delay is too long and more frequent updates are needed. However our results can give incentives to set up an additional registry of near real-time surveillance of SARI cases at the ICU. Our statistical analysis also has some limitations. Due to the weekly scope of the ILI data, we aggregated the ICU data on a weekly basis, losing detail as they are available on a daily basis. With regard to our chosen models to predict ILI, in the ideal situation stepwise variable selection is combined with 10-fold cross validation. Since automating this process is not possible in GEE, we first performed stepwise selection and then 10-fold cross validation on the seven remaining candidate models. Additionally, we used three years of data as training set to determine the best models, whereas a longer period would also have been an option but not necessarily better, since we continuously added data to the baseline data. Another limitation is that during the 2009–2010 pandemic, the ILI peaks were not detected or later. This means that our models were not sensitive to large or unexpected changes.

Apparently the association between ICU admissions and ILI in the general population can change greatly from season to season. ICU related SARI might occur at a very different rate (compared to symptoms in the general population) during a pandemic or unexpected seasons [9]. A probable explanation is that the influenza pandemic caused by the A(H1N1)pmd09 virus targeted another patient population than the previous epidemics with severe illness in younger patients, and fewer elderly with a severe infection. This could explain why increases in ICU admissions during the pandemic were later than usual. Additionally, the ICU data reflects only SARI cases. Therefore, we do not know if the ICU data reflect an influenza epidemic in the general population or possibly very different influenza dynamics in the ICU population alone.

### Conclusion

ICU data on respiratory infections was associated with ILI incidence, with highest association in the same week and in the week before and the week after. Increases in ICU data on average occur two days sooner and for a longer time period than increases in ILI. ICU data thus contains additional information on ICU related SARI cases during a specific influenza epidemic. Predicting influenza epidemics one, two or three weeks ahead in the general population using ICU data was imprecise, reflected by the low PPVs and sensitivities. Thus, ICU data cannot improve the current surveillance method to detect influenza epidemics. Due to the association between both data sources, a next step is to investigate the possibility of using ICU data in combination with microbiological laboratory results for surveillance of severity of illness, and ICU capacity prediction when an (severe) influenza epidemic is present.
